# Glyoxylic acid overcomes 1-MCP-induced blockage of fruit ripening in *Pyrus communis L*. var. ‘D’Anjou’

**DOI:** 10.1038/s41598-020-63642-z

**Published:** 2020-04-27

**Authors:** Seanna L. Hewitt, Rishikesh Ghogare, Amit Dhingra

**Affiliations:** 1 Molecular Plant Sciences Graduate Program, Washington State University, Pullman, WA USA; 2 Department of Horticulture, Washington State University, Pullman, WA USA

**Keywords:** RNA, Biochemistry, Plant sciences, Plant physiology

## Abstract

1-methylcyclopropene (1-MCP) in an ethylene receptor antagonist that blocks ethylene perception and downstream ripening responses in climacteric fruit imparting a longer shelf life. However, in European pear, the application of 1-MCP irreversibly obstructs the onset of system 2 ethylene production resulting in perpetually unripe fruit with undesirable quality. Application of exogenous ethylene, carbon dioxide and treatment to high temperatures is not able to reverse the blockage in ripening. We recently reported that during cold conditioning, activation of alternative oxidase (AOX) occurs pre-climacterically. In this study, we report that activation of AOX via exposure of 1-MCP treated ‘D’Anjou’ pear fruit to glyoxylic acid triggers an accelerated ripening response. Time course physiological analysis revealed that ripening is evident from decreased fruit firmness and increased internal ethylene. Transcriptomic and functional enrichment analyses revealed genes and ontologies implicated in glyoxylic acid-mediated ripening, including AOX, TCA cycle, fatty acid metabolism, amino acid metabolism, organic acid metabolism, and ethylene-responsive pathways. These observations implicate the glyoxylate cycle as a biochemical hub linking multiple metabolic pathways to stimulate ripening through an alternate mechanism. The results provide information regarding how blockage caused by 1-MCP may be circumvented at the metabolic level, thus opening avenues for consistent ripening in pear and possibly other fruit.

## Introduction

Every year, 1.6 billion tons of food goes to waste. This is about one-third of the food that is produced for human consumption^1^. Unpredictable ripening of fruit is one of the main causes of loss after harvest. This is particularly true of climacteric fruits, which are characterized by a spike in ethylene biosynthesis, known as system 2 (S2) ethylene production, and a concomitant burst in respiration at the onset of ripening. The ethylene receptor antagonist 1-methylcyclopropene (1-MCP) is used to impart a longer shelf life by limiting the ethylene perception and activation of downstream ripening responses^2–4^.

Uniquely, in European pear fruit (*Pyrus communis*), 1-MCP treatment may irreversibly inhibit endogenous or system 2 ethylene production and the respiratory climacteric^5,6^. Furthermore, exogenous ethylene application does little to affect the capacity of 1-MCP-treated pears to ripen^7,8^. This observation suggests that 1-MCP, which has been classically understood only in the context of its identity as an ethylene receptor antagonist^9^, seems to exert additional metabolic consequences. This presents a challenge to the U.S. pear industry, as 1-MCP pear fruit fail to ripen properly and do not achieve the desired buttery consistency, aromatics and flavor profile to meet consumer satisfaction standards^10^. Recent research has lent support to the concept of the differential effect of 1-MCP on ethylene biosynthetic pathways and signal transduction networks in different climacteric fruits, including peach, pear, apple, and tomato^11–13^. Attempts to metabolically reverse the ethylene antagonism of 1-MCP in pear and other climacteric fruit have not been reported previously.

In order to ripen, European pears require a genetically pre-determined amount of cold temperature exposure, known as conditioning^14^. Recent research revealed that Alternative Oxidase 1 (AOX1), the key protein in the cyanide resistant alternative respiratory pathway, is elevated significantly in transcript expression during the pre-climacteric stages of cold conditioning in ‘D’Anjou’ and ‘Bartlett’ pear^15^. Climacteric bursts in respiration have been observed in mango and apple fruit and are attributed in part to enhanced post-climacteric AOX activity^16,17^. It has been proposed that AOX and cytochrome c pathway activity may occur simultaneously in the ripening process, with the alternative respiratory pathway playing a greater role in the senescent processes following the climacteric burst^16,17^.

Based on the recent findings in pear, it appears that AOX may play a role in the pre-climacteric stage in fruits that require conditioning in order to ripen. Chemical genomics approaches targeting AOX for pre-climacteric ripening stimulation identified glyoxylic acid (GLA), the key metabolic intermediate in the glyoxylate cycle, as an activator of ripening in 1-MCP treated pear fruit^18^. The glyoxylate cycle is a shortcut in the tricarboxylic acid (TCA) cycle used by bacteria and plants for gluconeogenesis and carbohydrate synthesis via β-oxidation of fatty acids to acetyl coenzyme A (Ac-CoA)^19–21^. Thus far, the role of the glyoxylate cycle in ripening is largely unexplored, with no studies of metabolic override of 1-MCP ripening blockage reported in the present literature.

In this study, the hypothesis that GLA treatment will activate AOX expression and facilitate the identification of additional significant genes and gene networks that enable override of 1-MCP blockage of ripening was evaluated. Established indicators of ripening, including fruit firmness, total soluble solids, and internal ethylene production along with RNAseq, were analyzed over the course of ripening post-GLA treatment.

## Results and Discussion

**Figure 1:**
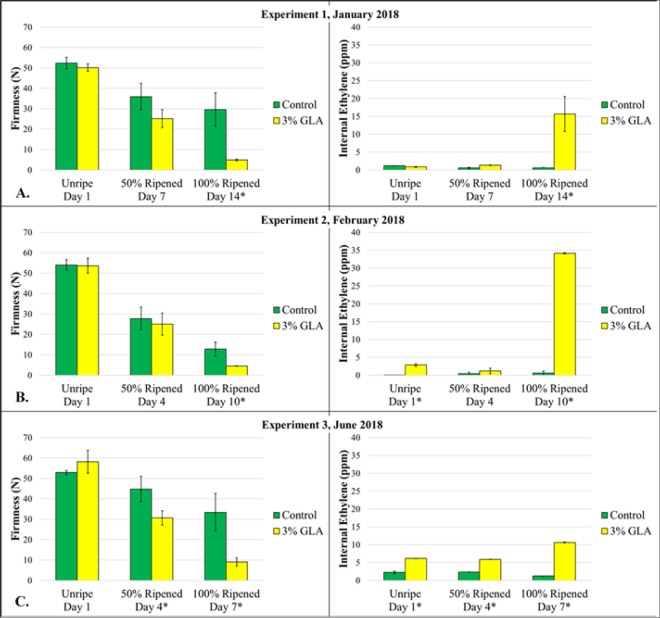
Firmness (in Newtons) and internal ethylene evolution (in parts per million) values for 1-methylcyclopropene (1-MCP) treated ‘D’Anjou pears following treatment with 3% glyoxylic acid (GLA) at Unripe, 50% Ripened, and 100% Ripened stages for experiments conducted in January 2018 (**A**), February 2018 (**B**), and June 2018 (**C**). Asterisks indicate a statistically significant difference at the respective time points (p < 0.05).

Glyoxylic acid treatment resulted in a significant decrease in firmness (p < 0.05) of 1-MCP treated ‘D’Anjou’ in comparison with the control in each of the three experiments conducted in 2018 (Fig. 1). Likewise, internal ethylene peaked significantly (p < 0.05) in the GLA-treated pears, a response characteristic of the S1-S2 ethylene transition and the ripening climacteric (Fig. 1). °Brix did not change significantly for either the treated or control pears throughout the duration of the experiment (Supplementary File 1). High-performance liquid chromatography (HPLC) mean glucose and fructose values were elevated in the 3% GLA treated fruit in comparison with the control despite lack of statistically significant difference between treatment and control groups. Furthermore, analysis of organic acids revealed increased mean malic acid (though not statistically significant) and highly increased citric acid production (p < 0.05) in the treated fruit vs the control fruit throughout the ripening time course (Supplementary File 2). An increase in citric acid implicates activation of the TCA cycle and respiration upon GLA treatment. Fruit tissues, including peel and flesh, sampled from the 3% GLA treated fruits and from the control fruits were used for a time-course transcriptomic analysis.

The duration that each experimental group of pear fruit was held in storage prior to GLA application must also be considered. Observations in this study indicated that the overall ripening time course, both in GLA-treated fruit and in control fruit, was shorter the longer the fruit had been in RA storage before the start of an experiment. This phenomenon necessitated modified sampling time points for each experiment described in the methods section. Treatment endpoint images and analysis of experiments conducted in 2017 that led to the optimization of the GLA treatment strategy can be found in Supplementary File 3.

### Transcriptomics

The RNAseq assembly generated 148,946 contigs (Supplementary File 4). Using the time course differential expression analysis feature in OmicsBox, 5,912 contigs were identified as being differentially expressed over time in the 3% GLA-treated fruit in comparison with the control in all three ripening experiments (Supplementary File 5). To better understand the mechanisms underlying GLA induction of pear fruit ripening, the expression of genes/contigs associated with the TCA cycle, glyoxylate cycle, lipid metabolism, AOX and mitochondrial electron transport, sugar/starch breakdown, and organic acid metabolism was analyzed (Table 1). Among the differentially expressed contigs (DECs) were ethylene metabolism-associated contigs and amino acid precursors, numerous ethylene response factors (*ERFs*), a pear *AOX* homolog (ubiquinol oxidase), TCA cycle and glyoxylate cycle-associated contigs, and lipid metabolism-associated contigs. Many of the identified DECs displayed heightened expression in the 3% GLA-treated fruit in comparison with the control fruit at the ‘Unripe’ stage, which was sampled immediately following the 16 hour GLA humidification treatment, indicating that this set of DECs directly responded to the application of GLA, but decreased in expression throughout the time course. Such genes with significantly heightened expression in the GLA- treated fruit immediately following treatment included: *AOX1*, several *ERF* transcripts, *copper-transporting responsive-to-antagonist 1* (*RAN1)*, *isocitrate dehydrogenase (IDH)*, lipoxygenase (LOX), and allene oxide synthase 1 (AOS1). DECs which displayed delayed transcriptional responses, which became evident at the ‘50% Ripened’ to ‘100% Ripened’ included: ethylene biosynthetic enzymes, 1-aminocyclopropane-1-carboxylate-synthase 1 (ACS1) and 1-aminocyclopropane-1-carboxylate oxidase 1 (ACO1), and citrate synthase, which displayed heightened expression in the treatment group in later stages of ripening.

**Table 1:** Summary of differentially expressed contigs discussed in the manuscript.

Associated Pathway	Gene/Contig Name	Abbreviation	Contig #	Contig Length (bp)	Significant Trend 3% Glyoxylic Acid (R > 0.8)
Alternative respiration	Ubiquinol oxidase, mitochondrial-like	AOX1	22331	1156	
Aspartate, cysteine, methionine metabolism	Aspartate aminotransferase, cytoplasmic	cytAAT	18640	503	Linear, Quadratic
Aspartate, cysteine, methionine metabolism	Bifunctional aspartate aminotransferase	BiAAT	10407	850	
Aspartate, cysteine, methionine metabolism	SAM synthase 2	SAM synthase 2	2250	1734	
Aspartate, cysteine, methionine metabolism	Probable pyridoxal 5’-phosphate synthase subunit PDX2	PDX2	6815	1473	
Aspartate, cysteine, methionine metabolism	Cystathionine gamma-synthase 1, chloroplastic	CγS1	9947	1598	
Ethylene biosynthesis	1-aminocyclopropane-1-carboxylate synthase	ACS1	61428	1380	Linear
Ethylene biosynthesis	ACC oxidase	ACO1	3326	595	
Ethylene perception	Copper-transporting ATPase RAN1	RAN1	20950	2399	Linear
Ethylene signaling/response	Ethylene-responsive transcription factor ERF073-like	ERF073-like	43790	243	
Ethylene signaling/response	Ethylene-insensitive 3-like 1 protein	EIN3-like 1	3680	454	Linear, Quadratic
Ethylene signaling/response	Ethylene-responsive transcription factor 3-like	ERF 3-like	11186	1079	
Ethylene signaling/response	Ethylene-responsive transcription factor 4 isoform X1	ERF4 isoform x1	20105	297	
Ethylene signaling/response	Ethylene-responsive transcription factor 5-like	ERF5-like	54832	1214	Linear
Ethylene signaling/response	Ethylene-responsive transcription factor ABR1-like	ERF ABR1-like	7129	1416	
Ethylene signaling/response	Ethylene-responsive transcription factor ERF096-like	ERF096-like	35082	706	
Ethylene signaling/response	Serine/threonine-protein kinase CTR1 isoform X1	CTR1 isoform x1	20857	650	
Fatty Acid/Oxylipin metabolism	Lipoxygenase 1	LOX1	4033	2690	
Fatty Acid/Oxylipin metabolism	Allene oxide synthase 1	AOS1	3632	1639	
Fatty Acid/Oxylipin metabolism	Allene oxide cyclase 4, chloroplastic	AOC4	2502	979	Quadratic
Fatty Acid/Oxylipin metabolism	Probable linoleate 9S-lipoxygenase 5	Linoleate 9S-LOX 5	15488	3275	Linear
Fatty Acid/Oxylipin metabolism	Linoleate 13S-lipoxygenase 3–1, chloroplastic-like	Linoleate 13S-LOX 3–1	10268	2980	
Glyoxylate cycle	Citrate synthase, glyoxysomal	gCS	3891	1784	
Glyxolysis/Gluconeogenesis	Phosphoenolpyruvate carboxylase kinase 1-like	PEPCK1-like	10747	754	
Glyxolysis/Gluconeogenesis	ATP-dependent 6-phosphofructokinase 3-like	PFK3-like	19400	904	
Glyxolysis/Gluconeogenesis	Fructose-1,6-bisphosphatase, cytosolic	F-1,6-Bpase	42519	255	Quadratic
TCA cycle	Fumarate hydratase 1, mitochondrial	mFH1	6653	845	Linear
TCA cycle	Isocitrate dehydrogenase [NADP]	IDH	7737	2131	
TCA/Glyoxylate cycle	Citrate synthase, mitochondrial	mCS	1515	418	
TCA/Glyoxylate cycle	Malate dehydrogenase, mitochondrial	mMDH	9623	2234	Linear

Information includes associated pathway, full and abbreviated names, contig number (corresponding to sequences, annotations, and expression values in Supplementary File 5), length, and an indication of significant trends.

### Glyoxylic acid activation of AOX

**Figure 2:**
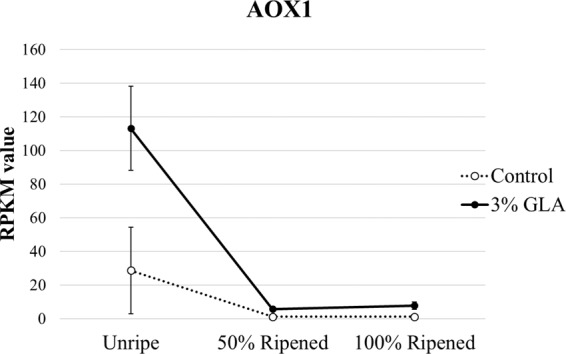
Normalized mean expression values of alternative oxidase 1 (AOX1) homolog during time-course sampling following 16-hour glyoxylic acid (GLA) treatment. AOX1 was differentially expressed over time in the 3% GLA-treated fruit than in the control fruit (p < 0.05), displaying significantly elevated expression at the Unripe stage, immediately following 16-hour GLA treatment.

AOX has been implicated previously in ethylene biosynthetic processes, the respiratory climacteric, and ripening. Immediately following treatment with GLA, *AOX* transcript abundance was higher, with its expression decreasing over time in both treatment and control 1-MCP fruit but remaining consistently higher in the GLA treatment group (Fig. 2). This expression trend is reminiscent of the pre-climacteric maxima of AOX transcription in non-1-MCP treated pear fruit that had undergone full conditioning^15,22^. AOX pathway activity has been shown to contribute to the respiratory climacteric during ripening in tomato, papaya, and mango^16,23–25^. While GLA activated *AOX*, the results of this transcriptomic analysis suggest that AOX activity in 1-MCP treated pear fruit is potentially regulated in a feed-forward manner as a result of carbon metabolism upstream^23,24,26^. This alternative respiratory stimulation may partially account for the enhanced respiratory response and ripening of 1-MCP treated pear^18^.

### Gluconeogenesis and glycolytic flux balance

The glyoxylate cycle is directly linked to the production of glucose via gluconeogenesis in certain biological contexts such as fatty acid to sugar conversion during seedling germination^19^. Previously, the study of gluconeogenic enzymes in tomato and peach revealed that *phosphoenolpyruvate carboxykinase* (*PEPCK*) abundance increases during ripening of these fruit^27^. It has also been demonstrated that malate and citrate accumulate throughout fruit development and may be utilized both as substrates for respiration in the TCA cycle, as well as for gluconeogenesis during fruit ripening; however, 1-MCP slows the rate of organic acid to sugar conversion via gluconeogenesis which thereby delays ripening^28–30^. Thus, it was of interest to examine the expression of genes in the gluconeogenic pathway in addition to the glycolytic pathway.

**Figure 3:**
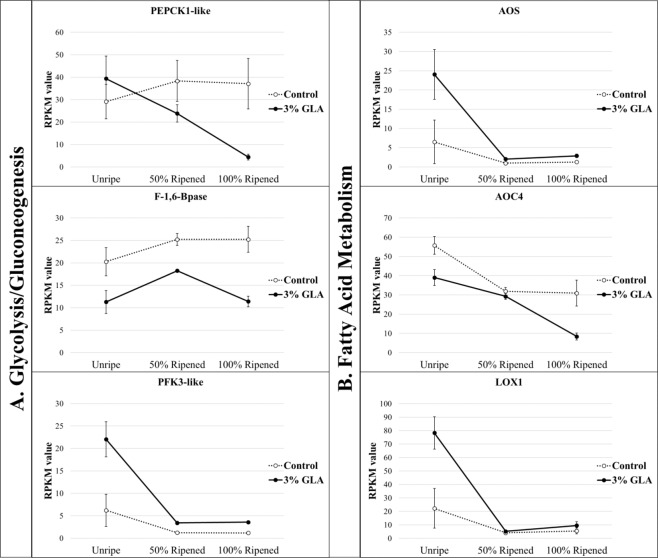
Normalized, mean expression values of genes encoding rate-limiting gluconeogenic and glycolytic enzymes (column **A**) and fatty acid and oxylipin metabolic enzymes (column **B**) during time-course sampling following 16-hour glyoxylic acid (GLA) treatment*. Phosphoenolpyruvate carboxykinase 1-like* (*PEPCK1-like*), *fructose-1,6-bisphosphatase* (*F-1,6-Bpase*), *phosphofructokinase 3-like* (*PFK3-like*), *allene oxide synthase* (AOS), *allene oxide cyclase 4* (*AOC4*), and lipoxygenase 1 (LOX1) were significantly differentially expressed over time in the 3% GLA-treated fruit versus the control fruit (p < 0.05).

The control 1-MCP fruit in this experiment displayed stable mean expression levels of *PEPCK* over the time course. In GLA treated 1-MCP fruit, mean expression levels of *PEPCK* were higher than in control immediately following treatment and gradually decreased over the time course, ending with very low expression levels in comparison with the control. *Fructose-1,6-bisphosphatase* (*FBPase*), which encodes an enzyme that plays a crucial additional role in gluconeogenic pathway, displayed elevated expression levels in control versus the treatment throughout the experimental time course (Fig. 3A). Conversely, *ATP-dependent 6-phosphofructokinase 3-like* (*PFK-like*) DEC exhibited dramatically elevated expression in the GLA-treated fruit immediately following treatment in comparison with the control fruit and decreased over time. PFK catalyzes the first committed step of glycolysis, which leads to the formation of ATP and pyruvate (Fig. 3A). The ATP generated through this process can catalyze other ripening related processes.

Taken together, these results suggest that in GLA-treated 1-MCP pear fruit, flux through the gluconeogenic pathway results in an additional allocation of carbon-based metabolites to the TCA cycle, potentially leading to over reduction of the mitochondrial electron transport chain, thereby necessitating increased AOX activity.

### Fatty acid metabolism and oxylipins

The glyoxylate cycle has an important role in β-oxidation of fatty acid under certain biological conditions, seedling germination being one of them, in which production of sugar from other organic substrates is of developmental importance^19,31^. The addition of GLA could potentially induce multiple metabolic pathways, thereby facilitating a link between fatty acid metabolism and GLA-induced ripening.

Fatty acid oxidation capacity typically is at its highest in pre-climacteric fruit and in fruit entering the climacteric stage^32^. Furthermore, a shift from mitochondrial oxidation of fatty acids to TCA cycle intermediates occurs in the climacteric transition (S1-S2 ethylene biosynthesis)^32^. In mango, the addition of glyoxylate- stimulated oxidation of fatty acids in a concentration-dependent manner^32^; it appears the same may be occurring in GLA treated 1-MCP pear fruit.

Oxidation of unsaturated fatty acids results in the generation of a class of lipophilic signaling molecules called oxylipins. The initial synthesis of these molecules occurs via conversion of polyunsaturated fatty acids to fatty acid hyperperoxides via lipoxygenase (LOX)^33^. The phytohormone jasmonic acid (JA) is among the most well-characterized oxylipins, as jasmonates, together with ethylene, are believed to play a role in the early stages of climacteric ripening^34,35^. Methyl jasmonate increases postharvest shelf life in Chilean strawberry (*Fragaria chiloensis*) by modulating the soluble solid to titratable acidity (TA) ratios as validated by assessments of quality chemical attributes and decay incidence^36^. JA has been shown to negatively regulate ripening in ‘Bartlett’ pears and is suggested to do so by working either independently or upstream of ethylene biosynthesis^37^. The first specific enzyme and rate-limiting point in JA biosynthesis is allene oxide synthase (AOS)^38^. Expression of *AOS* was significantly elevated in the GLA-treated fruit in comparison with the control 1-MCP fruit immediately following treatment, suggesting an increased flux through the JA biosynthesis pathway, which could potentially aid in further stimulation of an ethylene response in the treated fruit (Fig. 3B). In addition to *AOS*, several lipoxygenases were also significantly elevated in expression in the treatment versus the control fruit (some at the beginning and others at the end of the time course), indicating the commitment of fatty acid breakdown products to oxylipin metabolism (Fig. 3B). Based on these observations, it appears that fatty acid metabolism contributes both to a hormonal response and to increased pools of carbon-metabolites available for TCA cycle, thereby contributing to the multifaceted ripening response stimulated by GLA.

### TCA and glyoxylate cycle metabolism

**Figure 4:**
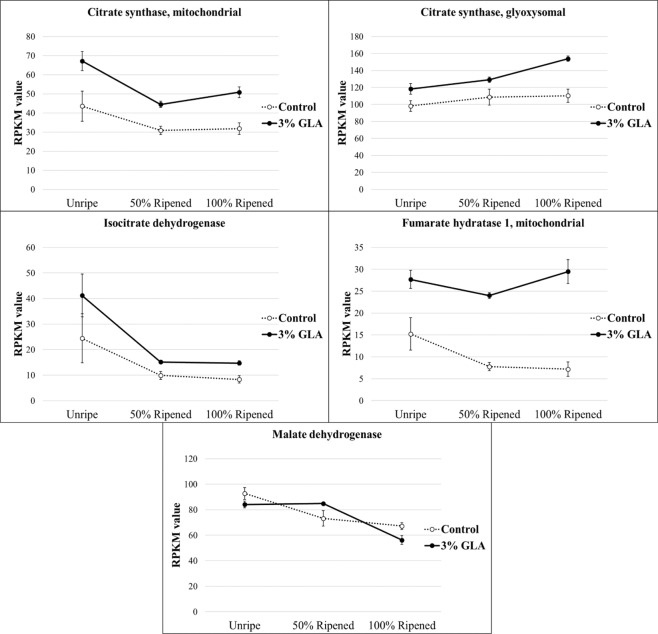
Normalized, mean expression values of genes encoding TCA/glyoxylate cycle enzymes during time-course sampling following 16-hour glyoxylic acid (GLA) treatment. Mitochondrial *citrate synthase* (*mCS*), glyoxysomal *citrate synthase* (*gCS*), *isocitrate dehydrogenase* (*IDH*), *mitochondrial fumarate hydratase* (*mFH*), and *malate dehydrogenase* (*MDH*) were significantly differentially expressed over time in the 3% GLA-treated fruit versus the control fruit (p < 0.05).

The glyoxylate cycle is primarily compartmentalized in the peroxisome; however, two of the five enzymes that comprise the cycle are cytosolic; glyoxysomal citrate synthase (gCS), glyoxysomal isocitrate lyase (gICL), glyoxysomal malate synthase (gMS), cytosolic aconitate hydratase (ctAC), and cytosolic malate dehydrogenase (ctMDH). Malate is an intermediate in both glyoxylate and mitochondrial TCA cycles and has been suggested to play a role as an important regulatory metabolite during ripening^39,40^. This important organic acid is involved in many other processes besides the glyoxylate cycle and respiratory metabolism, and therefore, membrane transport is required to shuttle this substrate into other subcellular compartments^41^. Glyoxylate cycle enzyme encoding malate synthase gene expression has been detected specifically in ripening tissue, but this expression varied by ripening stage and has been suggested that expression changes could be stimulated by exogenous ethylene^42,43^. Alteration in the supply of malate has been shown to affect postharvest storage quality^40^. Like malate, citrate is an intermediate in both TCA and glyoxylate cycles that accumulates in the pre-climacteric stages and is metabolized during ripening and is one of the most prevalent metabolites contributing to flavor and titratable acidity during fruit development^32^. Contigs corresponding to glyoxysomal and mitochondrial citrate synthase enzymes differed significantly in expression over time in GLA-treated 1-MCP fruit versus the control 1-MCP fruit, with increased expression of both observed in the treatment. Additional contigs, corresponding to TCA cycle enzymes fumarate hydratase and isocitrate dehydrogenase were also elevated in expression in GLA-treated fruit (Fig. 4). It implicated, based on the expression of TCA/glyoxylate cycle genes, that GLA could directly affect the flux through these pathways.

### Glyoxylic acid link to ethylene biosynthesis

Methionine cycling, and therefore ethylene production is a process that operates concomitantly with respiration and is co-dependent upon the TCA/glyoxylate cycle compound, oxaloacetate (OAA). In addition to serving as a substrate for the formation of citrate, OAA may be converted into intermediates for other metabolic processes^44^. The link between the glyoxylate and TCA cycle and ethylene begins with the conversion of OAA to aspartate via aspartate aminotransferase (AAT) or by bifunctional aspartate/prephenate aminotransferase (PT-AAT/PAT). Aspartate is then converted to homoserine, cystathionine, homocysteine, and then to methionine. Methionine is converted to S-adenosyl-L methionine (SAM) catalyzed by SAM synthetase, forming the precursor to 1-aminocyclopropane-1-carboxylic acid (ACC) and ethylene biosynthesis. The enzymes involved in these conversions depend on the co-factor pyridoxal-5’-phosphate (PLP), the production of which is catalyzed by pyridoxine synthases (PDX1 and PDX2)^45,46^. The heightened expression of genes in the path from OAA to the production of ACC in ethylene biosynthesis in the GLA-treated fruit in comparison with the control 1-MCP fruit suggests that the ripening compound elicits effects that upregulate this pathway via a yet uncharacterized mechanism. *AAT* expression was similar in the treatment and control at the experimental start and endpoints; however, its expression was significantly higher at the 50% Ripened stage (Supplementary File 6). *AAT/PAT* expression was highest in the GLA-treated fruit immediately following treatment, decreasing throughout the time course, but all the while remaining expressed at higher levels than the control fruit. Cystathionine gamma-synthase (*CγS)* expression remained highest in the GLA treatment over time than the control fruit. The expression pattern of *AAT/PAT*, *CγS*, and *SAM* as well as the cofactor producing *PDX2*, followed a nearly identical pattern, with the highest expression immediately following treatment and decreasing expression throughout the time course (Supplementary File 6). This methionine may feed directly into the methionine cycle, thereby resulting in increased ethylene production when OAA is produced abundantly. The observed changes in expression patterns suggest that increased flux through the glyoxylate/TCA cycles may lead to increased production of OAA, some of which is shunted into the methionine biosynthesis pathway and can be consequently converted to ethylene. Methionine cycling is an ATP-dependent process and in situations where respiration is interrupted, as is in the case of 1-MCP treatment, this fundamental process cannot occur. Activation of the glyoxylate cycle in fruit subjected to respiratory inhibition is the potential source for the ATP necessary for continued methionine cycling and, therefore, ethylene biosynthesis.

**Figure 5:**
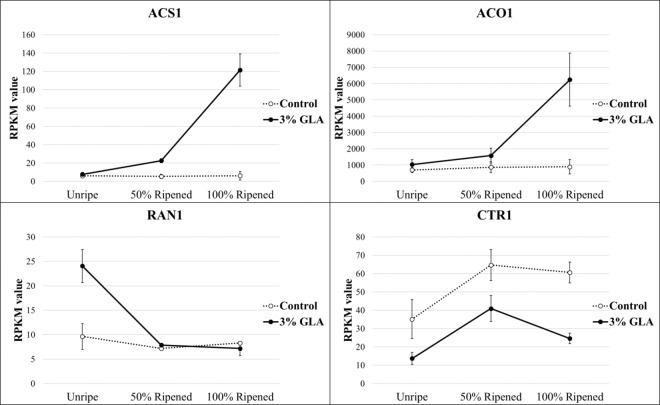
Normalized, mean expression values of genes involved in ethylene biosynthesis and perception during time-course sampling following 16-hour treatment with glyoxylic acid (GLA). *1-aminocyclopropane-1-carboxylate synthase 1* (*ACS1*), *1-aminocyclopropane-1-carboxylate oxidase 1* (*ACO1*), *copper transporting ATPase responsive-to-antagonist 1* (*RAN1*), and *constitutive-triple-response 1* (*CTR1*) were significantly differentially expressed over time in the treatment group versus the control (p < 0.05).

Significant changes in the expression of DECs associated with ethylene biosynthesis, perception, and signaling were identified throughout the ripening time course following GLA treatment, including those corresponding to ethylene biosynthetic genes *ACO1* and *ACS1*, ethylene perception-associated *RAN1* and *constitutive-triple-response 1* (*CTR1)* (Fig. 5), and numerous ethylene response factors (Supplementary File 7).

### Functional enrichment analysis

Gene ontology (GO) enrichment analysis was conducted using the differentially expressed contigs in the 3% GLA treatment group versus the control group as the test set and the annotated master assembly as the reference set, and the resulting enriched ontologies were reduced to the most specific ones (FDR < 0.05). The results provide complementary information to the differential expression analysis, with many of the over and underrepresented terms corresponding to biological processes and molecular functions associated with known and novel ripening related pathways (Supplementary File 8).

Overrepresented ontologies in the 3% GLA-treated fruit included: ‘fruit ripening’ and ‘response to chemical’. These are expected terms, considering that physiological changes indicative of ripening in response to chemical treatment were observed in the treated fruit. Additional overrepresented GO terms included those associated with phytohormone metabolism (‘1-aminocyclopropane-1-carboxylate biosynthetic process’, ‘hormone metabolic process,’ and ‘regulation of hormone levels’), ‘aspartate family amino acid biosynthetic process’, metabolism of sulfur compounds (‘sulfate transport, ‘sulfur compound metabolic process’), organic acid metabolism (‘carboxylic acid catabolic process’, ‘oxoacid metabolic process’), lipid metabolism (‘lipid metabolic process’, ‘oxylipin biosynthetic process’). These findings coincide with the results of the differential expression analysis where contigs associated with ethylene biosynthesis and signaling, organic acid metabolism, and lipid metabolism displayed significant differential expression (Figs. 3–5), providing support for crosstalk between these seemingly independent pathways during GLA-mediated ripening. Furthermore, the results of this analysis again implicate AOX as an important factor in ripening. Sulfur metabolism-associated terms were overrepresented, and sulfur has previously been implicated in the pre-climacteric activation of the AOX respiratory pathway and ripening in cold-conditioned pear fruit^18^. Interestingly, ‘response to cold,’ ‘cellular oxidant detoxification’ and ‘antioxidant activity’ were also enriched. While these experiments were conducted at ambient temperature, cold conditioning is required for pear fruit to ripen^5^, and recent studies of cold-induced ripening of pear implicate the involvement of AOX for crosstalk with ethylene and scavenging of reactive oxygen species (ROS), thereby alleviating oxidative damage under external stress conditions^15,47^. Enrichment of these ontologies may indicate that similar mechanisms are activated in both cold and GLA- induced ripening, with both instances involving AOX activity. It raises an interesting possibility that GLA might be able to, in part, replace the requirement of cold conditioning in pear.

Underrepresented GO terms include those associated with maintenance of DNA and chromosomal integrity and organization (‘DNA-dependent DNA replication,’ ‘DNA repair,’ ‘DNA conformation change,’ ‘chromosome modification,’ ‘chromatin organization,’ and ‘histone modification’) (Supplementary File 8). The decreased representation of these functions in the GLA-treated test set, which exhibited physiological and transcriptomic indications of more rapid senescence, indicates that compositional organization decreases, and cellular entropy increases as ripening proceeds. These processes are directly correlated to cell wall softening, and other processes of decompartmentalization in the ripening fruit. Backtracking from the GO enrichment data to corresponding differentially expressed genes may provide additional targets for ripening regulation in several related pathways.

### Glyoxylic acid as a metabolic hub

**Figure 6:**
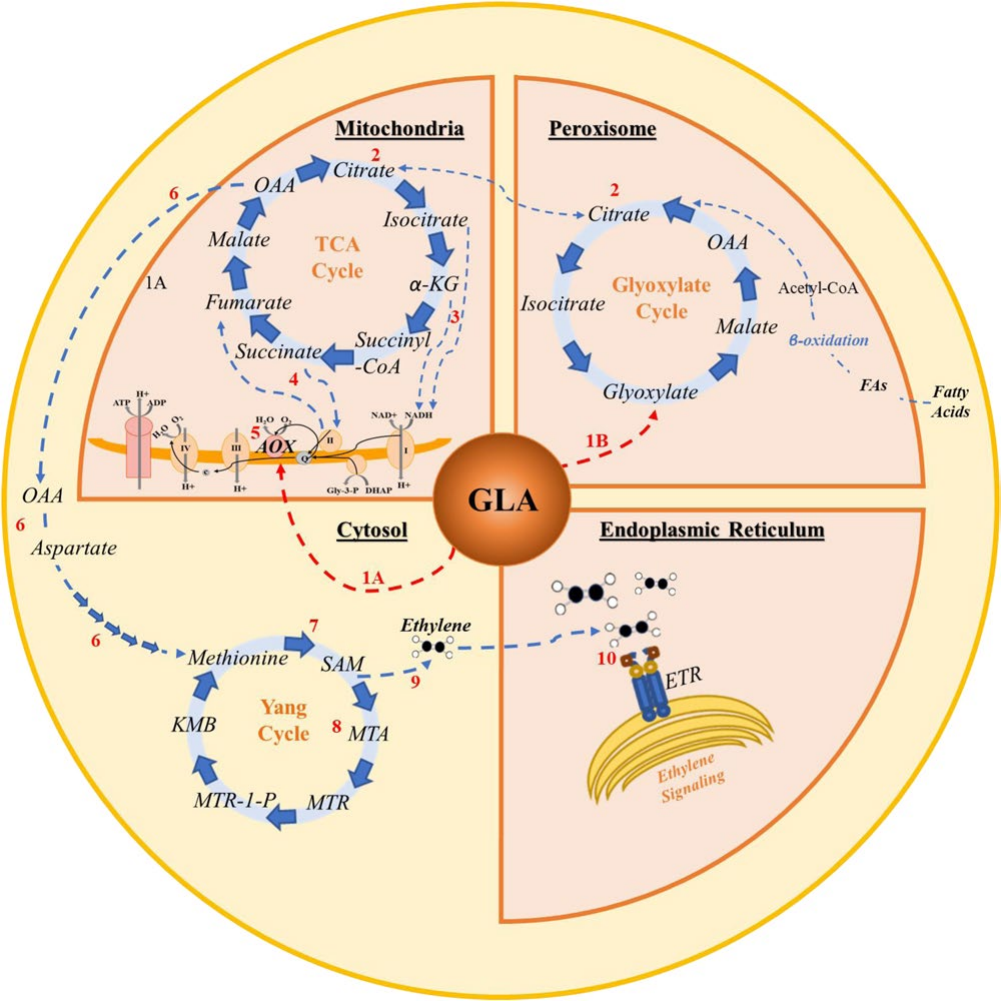
Proposed model for glyoxylic acid (GLA) as a metabolic hub connecting ripening associated pathways in the peroxisome (glyoxylate cycle and fatty acid β-oxidation), mitochondria (tricarboxylic acid (TCA) cycle and cytochrome c (CYTc) pathway), cytoplasm (methionine cycle and ethylene biosynthesis), and endoplasmic reticulum (ethylene perception and signaling). (1) Putative sites for direct activation of alternative oxidase (AOX) are indicated by the red dashed arrows. 1A-Glyoxylate has previously been shown to activate AOX activity in Arabidopsis^48^ directly, and GLA is hypothesized to elicit similar AOX activation in other systems, indicated by a red dashed arrow. 1B-Addition of GLA increases pools of metabolic intermediates in the glyoxylate cycle, resulting in increased flux through the cycle. (2) Production of malate and its subsequent conversion to oxaloacetate (OAA), perpetuates the glyoxylate cycle, producing intermediates shared by the TCA cycle, including those which can be shuttled between intracellular compartments (e.g., citrate). Thus, GLA leads to increased flux through both glyoxylate and TCA cycles. (3) The oxidative decarboxylations of isocitrate and α-ketoglutarate in the TCA cycle result in the transfer of high-energy electrons to NADH, which is transported to the mitochondrial CYTc pathway. (4) Conversion of succinate to fumarate in the TCA cycle results in the transfer of additional high energy electrons to complex II of the CYTc pathway. (5) The resulting increased flux of electrons necessitates the activation of AOX to prevent CYTc over reduction. (6) In addition to respiratory activation, the TCA and glyoxylate cycles share the common intermediate OAA, which can be converted to aspartate and subsequently to the precursors of methionine. (7) Methionine, a precursor for ethylene biosynthesis, is converted to S-adenosyl-L-methionine (SAM). From SAM, methionine is either (8) recycled via the Yang cycle, or (9) converted directly into ethylene. (10) Ethylene binds to endoplasmic reticulum (ER) bound receptors (ETRs), initiating the ethylene signal cascade and downstream ripening-associated responses.

The findings of this study suggest that the propensity of GLA to stimulate a ripening response in pear may result from the centrality of the glyoxylate metabolism to several critical biochemical pathways, as evidenced by the elevation in expression of genes encoding critical rate-limiting enzymes in glycolysis, fatty acid metabolism, aspartate/cysteine/methionine metabolism, TCA cycle, and the AOX respiratory pathway immediately following GLA treatment (Fig. 6). Interestingly, while such closely associated processes with the glyoxylate cycle appear to be stimulated upon GLA administration, differential expression of the unique glyoxylate cycle enzymes malate synthase and isocitrate lyase was not observed. This finding necessitates further protein expression-based work but could also indicate that following application, GLA is directly converted into other substrates that can be used for respiration and ethylene biosynthesis—testing the effects of continual administration of GLA at low levels throughout a designated ripening period could shed light on this hypothesis. Such an approach has already been successfully employed with other compounds, including 1-MCP^6^. Additionally, investigating the potential stimulatory effects of exogenously applied TCA and glyoxylate cycle intermediates would add further insight to the understanding of the way in which these pathways and their metabolic intermediates affect critical developmental processes like ripening.

## Conclusion

This study allowed for the identification of interacting networks of genes and pathways that are involved in GLA-mediated activation of AOX and the metabolic override of ripening blockage in pear caused by 1-MCP. Based on prior knowledge of the glyoxylate cycle, its intermediates, and its regulation, it is hypothesized that its mode of action in ripening induction occurs via one or more of the following processes: direct activation of AOX, induction of fatty acid oxidation, provision of organic acid as respiratory substrate, indirect TCA cycle stimulation, or induction of gluconeogenesis^31,32,48–51^ (Fig. 6). Results of time course differential expression analysis of GLA-treated ‘D’Anjou’ 1-MCP pear fruit compared to control 1-MCP fruit lend support to this hypothesis and have provided additional molecular targets for fine-tuned ripening regulation of European pear and other climacteric fruits subjected to 1-MCP treatment.

Ripening in climacteric fruit has primarily been attributed to the biosynthesis of ethylene. It is clear, however, that the nuances of ripening extend far beyond ethylene production and respiration^52^. Due to complex requirements for S1-S2 transition in European pear, the fruit may serve as a useful system in which to explore the vagaries of the ripening process in climacteric and non-climacteric fruit^52^. This study addresses a knowledge gap in the ripening process in pear, epitomized by the inability of 1-MCP treated fruit to regain ripening capacity naturally and paralleled with the discovery that GLA can overcome the 1-MCP blockage. Exploring the potential role of glyoxylate as a metabolic hub, its link to ethylene biosynthesis, fatty acid metabolism, glycolysis, and activation of the AOX respiratory pathway in the context of 1-MCP inhibited ripening, provides further insight into the mechanism of ripening in European pear and other fruit. It also enriches understanding regarding how ripening control may be better achieved through 1-MCP inhibition and subsequent stimulation of ripening using natural metabolic intermediates.

The role of GLA in the stimulation of ripening and the potential genes and pathways implicated in this process provide additional molecular targets for fine-tuned regulation of ripening and contribute to a body of work in which genetic markers associated with sugars, acids, volatiles, and ripening, in general, are being mapped^53,54^. As GLA is a natural plant metabolite and has previously been used as a commercial food preservative^15,55^, it is feasible to introduce it as a ripening tool in the tree fruit industry. Translation of this ripening toolset—1-MCP inhibition and GLA activation—to other crops in the future is expected to provide the opportunity to inhibit ripening at harvest, store fruit for the desired duration or ship it to domestic or international locations. Ripening can be reactivated in a planned and predictable manner, thereby improving consumer satisfaction and ultimately improving postharvest sustainability by reducing the massive waste associated with unpredictable ripening of fruit.

## Materials and Methods

### Acquisition of ‘D’Anjou’ pear fruit

Mature, late-season ‘D’Anjou’ pears were obtained from Blue Star Growers (Cashmere, WA) in the Winter/Spring of 2018. Before the acquisition, pears had been treated via 24-hour fumigation at the grower’s packinghouse with 130ppb 1-MCP and retained in controlled atmosphere (CA) storage at 1 °C, per industry standard practice. Following the acquisition, pears were transferred to a 1 °C Regular Atmosphere (RA) storage room at the Washington State University Johnson Hall postharvest facility for approximately one week before initiation of the first experiment in January 2018. The fruit was held at RA prior to the start of second and the third experiment in February and June 2018, respectively.

### Ultrasonic humidification of ripening compound treatment solutions

Pears were transferred to non-airtight plexiglass tanks (dimensions 40 cm × 58 cm × 66 cm) fitted with an inlet for a tube connected to a Crane Ultrasonic humidifier (Crane USA). The bottom of each humidifier was lined with baker’s drying racks to allow for excess treatment liquid to drip to the bottom of the chamber during the humidification period. Humidifiers were loaded with treatment and control solutions of 3% GLA and deionized water, respectively (Supplementary File 9). Depending on the number of samples to be taken throughout the experiment, between 32 and 64 pears were treated in each chamber. The pears were treated for a total of 16 hours overnight with their designated ripening compound or control solution. Following GLA treatment, pears were maintained at ambient temperature (20 °C) and RA conditions for the remainder of the experiment.

### Tissue sampling

**Figure 7:**
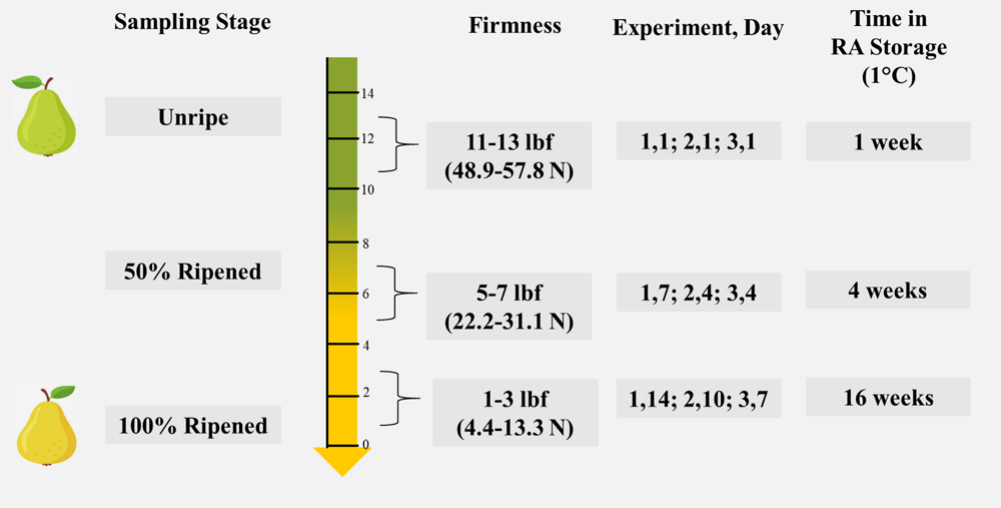
Sampling time-course and experimental replicate specific information for ‘D’Anjou’ pear fruit used in each glyoxylic acid (GLA) experiment. The fruit was classified as ‘Unripe’ on Day 1, immediately upon removal from GLA or control humidification chambers, when firmness between 11–13 lbf (48.9–57.8 Newtons). The classification as ‘50% Ripened’ was given to fruit at the sample point where average firmness of 3% GLA-treated fruit was between 5–7 lbf (22.2–31.1 Newtons). The fruit were classified as ‘100% Ripened’ when the firmness of the 3% GLA-treated fruit were between 1–3 lbf (4.4–13.3 Newtons). Time in RA storage prior to GLA treatment of technical replicates is also indicated.

It was necessary to classify sampling points to be used for sequencing based on percent ripeness. This method of sample point determination has been previously employed in ripening studies^15,56^. The fruit was classified as ‘Unripe’ on Day 1, immediately upon removal from GLA or control humidification chambers, when the firmness was between 11–13 lbf (48.9–57.8 N). The classification as ‘50% Ripened’ was given to fruit at the sample point where average firmness of 3% GLA-treated fruit was between 5–7 lbf (22.2–31.1 N). The fruit were classified as ‘100% Ripened’ when the firmness of the 3% GLA-treated fruit was between 1–3 lbf (4.4–13.3 N) (Fig. 7). Sample analysis stages corresponding to 0, 50, and 100% Ripened for Experiment 1 (January-February 2018) were determined to be Day 1, Day 7 and Day 14; Experiment 2 (February 2018) Day 1, Day 4, and Day 10; and Experiment 3 (June 2019) Day 1, Day 4 and Day 7.

In each experiment, peel and flesh tissue were sampled from a 1 cm wide equatorial region of each of 4 fruit per treatment and time point, pooled, flash frozen in liquid nitrogen, and ground using a SPEX Freezer/Mill 6870 (Metuchen, NJ USA). Tissues were stored at −80 °C prior to RNA extraction and HPLC analysis.

### Internal ethylene gas chromatographic measurements

Ethylene gas was vacuum extracted from the inside of the fruit using a vacuum aspirator according to previously described methods^57^. One-quarter of four separate pear fruit per treatment was sliced into 4–5 pieces and then placed into an inverted funnel submerged at the base in previously de-gassed deionized water (Supplementary File 9). Headspace air was removed through a septum at the top of the funnel. Subsequently, the vacuum chamber was sealed, and the aspirator used to extract the gas from within the fruit. 0.5 mL headspace gas that collected in the neck of the inverted funnel was extracted in triplicate with 1 mL hypodermic syringes.

Gas samples (2 replicates per treatment, 1/4 pear sections from 4 fruits per replicate) were injected into a HP 5890 A gas chromatograph (Agilent, Avondale, PA, USA) with a flame ionization detector (FID) connected to a 0.53 mm × 15 m GS-Q-PLOT column (Agilent) to measure the ethylene composition of the extracted gas. Ethylene gas composition measurements were repeated every three days throughout each experimental time course. The mean and standard error for ethylene evolution was calculated for each sample time point.

### Firmness measurements

Firmness measurements were collected at each time point using four replicate pears from each treatment group. A GS-14 Fruit Texture Analyzer (GÜSS Instruments, South Africa) with an attached 8.0 mm probe set at 5.0 mm flesh penetration was used to measure firmness at 3 equidistant points around the equatorial region of each fruit following peel removal. Mean firmness value for each fruit was used for the final data assessment.

### °Brix

The soluble solid content was measured at each sample point by pooling approximately 0.5 mL extracted juice from four replicate fruit per treatment/control and quantifying *°Brix* using a handheld refractometer.

### HPLC analysis

100 mg of pulverized tissue from each sample was vortexed with 1 mL dH_2_O for 30 seconds. The particulate was removed using 25 mm 0.45 μm syringe filters (VWR) and filtered liquid transferred into HPLC vials (Thermo Scientific). Each sample was analyzed using Bio-Rad Aminex HPX-87H column (300 mm × 7.8 mm, 9 µm particle size; Bio-Rad Laboratories, Hercules, California, USA), attached to a Varian Pro Star 230 HPLC system (Varian Inc., California, USA) equipped with UV and Refractive Index (RI) detectors. The column and RI detector were maintained at 65 °C and 55 °C, respectively. For each sample, the injection volume was set at 10 µL and 0.005 M sulfuric acid solution was used as an eluent. The samples were eluted at a flow rate of 0.6 mL/min over 50 min. Organic acids (citric and malic) were detected using UV detector at 210 nm and sugars (glucose and fructose) were detected using the RI detector. Different organic acids and sugars were identified by comparing the retention time of the peaks with known individual standards (Sigma Aldrich, St. Louis, Missouri, USA) run under the same conditions. Quantification was achieved using the external standard method.

### Statistical analysis of physiological data

Analysis of variance (ANOVA) was conducted for ethylene, firmness, °Brix, and HPLC data within and across each of the experiments using SAS University Edition (SAS Institute Inc., Cary, NC) with time, and GLA as treatments.

### RNA extraction and sequencing

Total RNA was extracted from pulverized ‘D’Anjou’ peel and flesh tissue for each of the three technical replicates at ‘Unripe’, ‘50% Ripened’, and ‘100% Ripened’ stages following fruit tissue-specific DEPC-CTAB protocol^58^. RNA was quality checked on an agarose gel and was quantified using Nanodrop 2000 spectrophotometer (Thermo Scientific, Waltham, MA, USA). Following quality validation and quantification using a Life Technologies Qubit Fluorometer (Carlsbad, CA) and Agilent Bioanalyzer (Santa Clara, CA), RNA libraries were sequenced by BGI Hong Kong Tech Solution NGS Lab on an Illumina HiSeq. 4000 platform as 2 × 100 paired end reads.

### Transcriptome assembly

The 2 × 100 paired-end fastq files generated using Illumina HiSeq. 4000 were input into the CLC Bio Genomics Workbench (ver 6.0.1) (Aarhus, Denmark) for pre-processing and assembly. The CLC Create Sequencing QC report tool was used to assess quality. The CLC Trim Sequence process was used to trim quality scores with a limit of 0.001, corresponding to a Phred value of 30. Ambiguous nucleotides were trimmed, and the 13 5′ terminal nucleotides removed. Reads below length 34 were discarded. Overlapping pairs were merged using the’Merge Overlapping Pairs’ tool, and a subsequent de novo assembly was performed with all datasets. Parameters used in the assembly were as follows: Map reads back to contigs = TRUE, Mismatch cost = 2, Insertion cost = 3, Deletion cost = 0.4, Similarity Fraction = 0.95, Global Alignment = TRUE, Minimum contig length = 200, Update contigs = TRUE, Auto-detect paired distances = TRUE, Create list of un-mapped reads = TRUE, Perform scaffolding = TRUE. The de novo assembly resulted in the production of 148,946 contiguous sequences (contigs). Contigs with less than 2x coverage and those less than 200 bp in length were eliminated. For each individual dataset (treatment/replicate) the original, non-trimmed reads were mapped back to the master assembly subset. Default parameters were used, except for the length fraction, which was set to 0.5, and the similarity fraction, which was set to 0.9. Mapping resulted in the generation of individual treatment sample reads per contig. The master transcriptome was exported as a fasta file for functional annotation and the read counts for each dataset were exported for normalization with the Reads Per Kilobase per Million reads (RPKM) method^59^.

### Functional annotation

The master transcriptome fasta produced from the Illumina assembly was imported into OmicsBox 1.1.135 (BioBam Bioinformatics S.L., Valencia, Spain) for functional annotation of expressed contigs. Contig sequences were identified by a blastx alignment against the NCBI ‘Viridiplantae’ database with and e-value specification of 10.0E-3. GO annotation was assigned using the ‘Mapping’ and ‘Annotation’ features using default parameters to generate a functionally annotated master assembly^60^.

### Differential expression analysis

Temporally differentially expressed genes were identified using the time course, multi-series differential expression feature in the OmicsBox suite, which employs the maSigPro R package^61^. The FDR cutoff value was set to 0.05. The statistical analysis ensured that genes that did not meet the assumption of equal variances were eliminated from the analysis, which was particularly important given that the three experiments were performed at different times throughout the 2018 season. The DEGs and expression values were matched with their corresponding functional annotations (Supplementary File 5).

### GO enrichment analysis

Gene ontology (GO) enrichment analysis was conducted to determine over and underrepresented biological processes, molecular functions, and cellular components among the differentially expressed sequences using the OmicsBox Enrichment Analysis (Fisher’s Exact Test) function^60^ (Supplementary File 8). The annotated master transcriptome was used as the reference dataset, and the set of genes identified as differentially expressed over time in the treatment group versus the control group was used as the test dataset.

### qRT-PCR validation

Primers for qRT-PCR targeting seven differentially expressed genes in the ripening-related pathways discussed previously were designed using the NCBI Primer-BLAST tool^62^. 200 ng RNA for each sample was used to generate 1st strand cDNA using the Invitrogen VILO kit (Life Technologies, Carlsbad, CA USA). cDNA preparations were then diluted to 20 ng/uL. Final library concentrations were quantified using a Qubit fluorometer (Carlsbad, CA). qRT-PCR technical replicate reactions were prepared for each of the gene targets using the iTAQ Universal SYBR Green Supermix with ROX reference dye (BioRad, Hercules, CA) per the manufacturer’s protocols with 20 ng of template cDNA. In a Stratagene MX3005P, the following thermocycler profile was used: 95 °C initial disassociation for 2:30 minutes followed by 50 amplification cycles (95 °C for 30 s, 60 °C for 30 s, and 72 °C for 30 s) and a final, single-cycle phase to generate a dissociation curve (95 °C for 30 s, 57 °C for 30 s, and 72 °C for 30 s). The LinRegPCR tool was used to calculate the Cq values for each reaction^63,64^ (Supplementary File 10). Cq values, which were calculated from efficiency scores below 1.80 or above 2.20 were considered sufficiently low in confidence and were deemed unacceptable and were omitted from the analysis.

## Supplementary information

Supplementary Information.

Supplementary Information2.

Supplementary Information3.

Supplementary Information4.

Supplementary Information5.

Supplementary Information6.

Supplementary Information7.

Supplementary Information8.

Supplementary Information9.

Supplementary Information10.

Supplementary Information11.
