# Supplementary material for: Glyoxylic acid overcomes 1-MCP-induced blockage of fruit ripening in Pyrus communis L. var. ‘D’Anjou’
Source: Sci Rep. 2020 Apr 27;10:7084. doi: 10.1038/s41598-020-63642-z (PMC7184741; doi:10.1038/s41598-020-63642-z)
Supplement: Supplementary file 1 — Supplementary Information. [file 41598_2020_63642_MOESM1_ESM.docx]

**Glyoxylic acid overcomes 1-MCP-induced blockage of fruit ripening in *Pyrus communis L.* var. ‘D’Anjou’**

Seanna Louise Hewitt^1,2^, Rishikesh Ghogare^2^, and Amit Dhingra^1,2*^

^1^-Molecular Plant Sciences Graduate Program, Washington State University, Pullman, WA

^2^-Department of Horticulture, Washington State University, Pullman, WA

*Author to whom correspondence should be addressed: [adhingra@wsu.edu](mailto:adhingra@wsu.edu)

SUPPLEMENTARY FILES

**Supplementary File 1.** Soluble solid content of 1-MCP treated ‘D’Anjou’ pears following treatment with 3% GLA in February and June 2018 experiments.

**Supplementary File 2.** Mean HPLC profiles for glucose, fructose, citric acid, and malic acid for 2018 1-MCP ‘D’Anjou’ pears treated with 3% GLA solution.

**Supplementary File 3.** Additional results from GLA experiments and pH experiments conducted in the 2017 and 2018 pear seasons.

**Supplementary File 4.** Master transcriptome assembly fasta file for control and 3% GLA treated 1-MCP ‘D’Anjou’ pear fruit.

**Supplementary File 5.** Mean RPKM values, standard error, and time course differential expression information for GLA-treated and control ‘D’Anjou’ pear fruit.

**Supplementary File 6.** Genes involved in metabolism of amino acids leading into precursors of ethylene biosynthesis that were differentially expressed in the 3% GLA-treated 1-MCP ‘D’Anjou’ pear fruit versus control.

**Supplementary File 7.** Ethylene response factor encoding genes that were differentially expressed in 3% GLA-treated 1-MCP ‘D’Anjou’ pear fruit versus control.

**Supplementary File 8.** Enriched gene ontologies for GLA-treated pear fruit versus control pear fruit during experimental time course.

**Supplementary File 9.** Images of GLA ultrasonic humidification system, internal ethylene gas extraction method, and CO_2_ evolution monitoring system.

**Supplementary File 10.** Quantitative RT-PCR results with LinRegPCR output and calculated expression values.
