# Supplementary material for: Glyoxylic acid overcomes 1-MCP-induced blockage of fruit ripening in Pyrus communis L. var. ‘D’Anjou’
Source: Sci Rep. 2020 Apr 27;10:7084. doi: 10.1038/s41598-020-63642-z (PMC7184741; doi:10.1038/s41598-020-63642-z)
Supplement: Supplementary file 4 — Supplementary Information4. [file 41598_2020_63642_MOESM4_ESM.docx]

**Results**

Experiments using glyoxylic acid (GLA) solutions of various concentrations and pHs were conducted during the 2017 and 2018 pear seasons (Supplementary Table 1).  Three experiments conducted in 2017 pear season allowed for identification of optimal GLA treatment solution and sampling time course to elicit ripening responses. Based on the observations that GLA at its native pH of 2.3 elicited the most prominent ripening response, resulting in significantly decreased firmness, increased internal ethylene production, and CO_2_ evolution over time in comparison with the control, 3% GLA solutions at native pH and pH values titrated to 4 and 6 were selected as the treatment solutions to be tested in the 2018 experiments.

The 2017 experiments revealed that as the concentration of GLA applied to fruit increased in the 0 to 3% range, more dramatic visual responses were observed, particularly related to the aesthetic quality and textural composition of the fruit. Increased incidence of peel tissue burning was observed with higher GLA concentrations (Supplementary Figure 1). Fruit physiology was also altered as a result of ripening compound, with fruit treated with GLA at native pH (2.3) displaying the most dramatic decrease in firmness, increase in internal ethylene and increase in CO_2_ evolution over time, in comparison with fruit treated with GLA solutions titrated to neutral pH and the control solution (Supplementary Figure 2a-c) or with solutions of 1% and 2% GLA (Supplementary Figure 3a-c).

To test whether acidity of GLA solutions was responsible for induction of ripening in the fruit, and to observe the effects of neutrally shifted pH on ripening, several pH titrations were employed in a trial with 1% GLA solutions at native pH and pH 6, along with a control solution of D-isoascorbic acid. The final compound was chosen as an acidity control because it is the isomer of ascorbic acid that is not metabolized by plants, and therefore serves to represent the effects of acidic compound without affecting metabolism of the fruit in other ways^1^. Treatment with D-isoascorbic acid yielded results similar to the those of the control, in which the fruit did not display a significant ripening response in comparison with either of the 1% native pH solution or the 1% pH 6 solution, both of which displayed decreases in firmness and increases in internal ethylene production during the ripening experiments (Supplementary Figure 4a-b). The results of the preliminary trials conducted in 2017 suggested that the acidic nature of the GLA treatment solutions is, in part, responsible for accelerated ripening of the fruit, potentially via alteration of the capacity of enzymatic reactions to take place; however, application a non-metabolizable acidic compound alone (D-isoascorbic acid) was not sufficient for induction of ripening, although it did result in notably less superficial damage to the outer surface of the fruit.

2018 pH experiments, although conducted at the same time as the control and 3% GLA experiments used for transcriptome sequencing, were not reported in the manuscript. Results of those studies are reported here in addition to those of the 2017 experiments (Supplementary Figures 5-7).

**Methods**

**Firmness, °Brix, internal ethylene GC measurements, and statistical analysis**

Measurements of these parameters were conducted as described in the manuscript.

**Monitoring of carbon dioxide evolution**

Following 16-hour humidification treatment, GLA-treated and control pears were weighed and sealed into continuous air flow chambers (four replicates of 4 fruit per replicate). Airflow to each chamber averaged ca. 83 mL min −1 of CO2-free air. CO2 was automatically sampled at 4-hour intervals. Fruit respiration rates were determined by calculating mean CO2 evolution per kilogram of fruit at each sample time using the previously described methods and sampling system^2^

**Supplementary Table 3.1** Informational table detailing 1-MCP ‘D’Anjou’ pear GLA experiment date ranges, treatments applied during each experiment, and the growers from which pears were obtained.

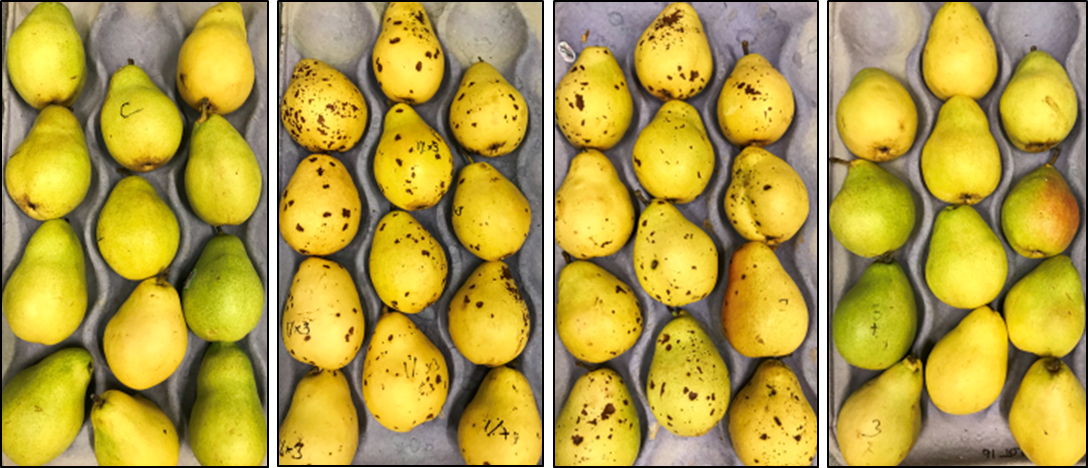


**
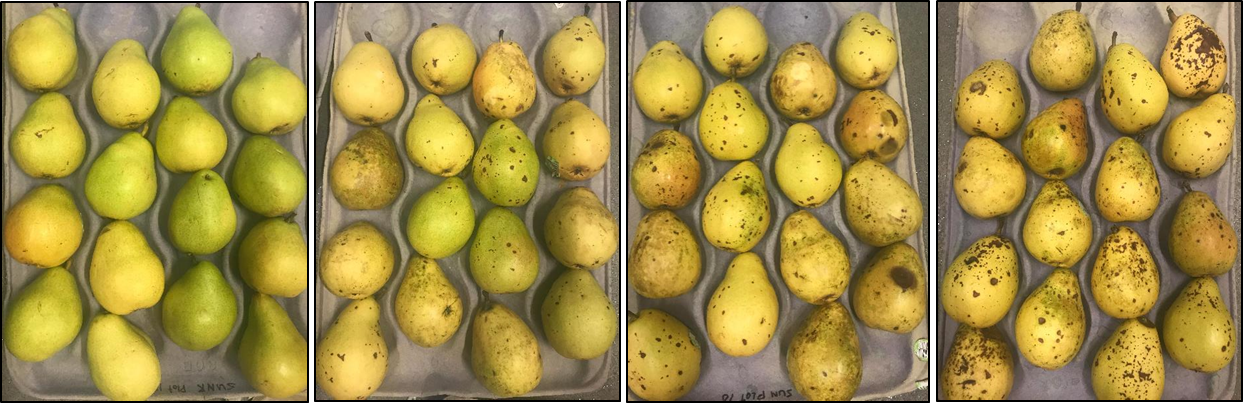
**

**Supplementary Figure 3.1.** Images taken on final day of a GLA concentration experiment. Top— ‘D’Anjou’ pears at day 14 following GLA/pH treatments. Left to right: control; 1% GLA (three applications); 3% GLA (native pH); 3% GLA pH 6. Bottom— ‘D’Anjou’ pears at day 14 following GLA treatments. Left to right: Control, 1% GLA, 2% GLA, 3% GLA.


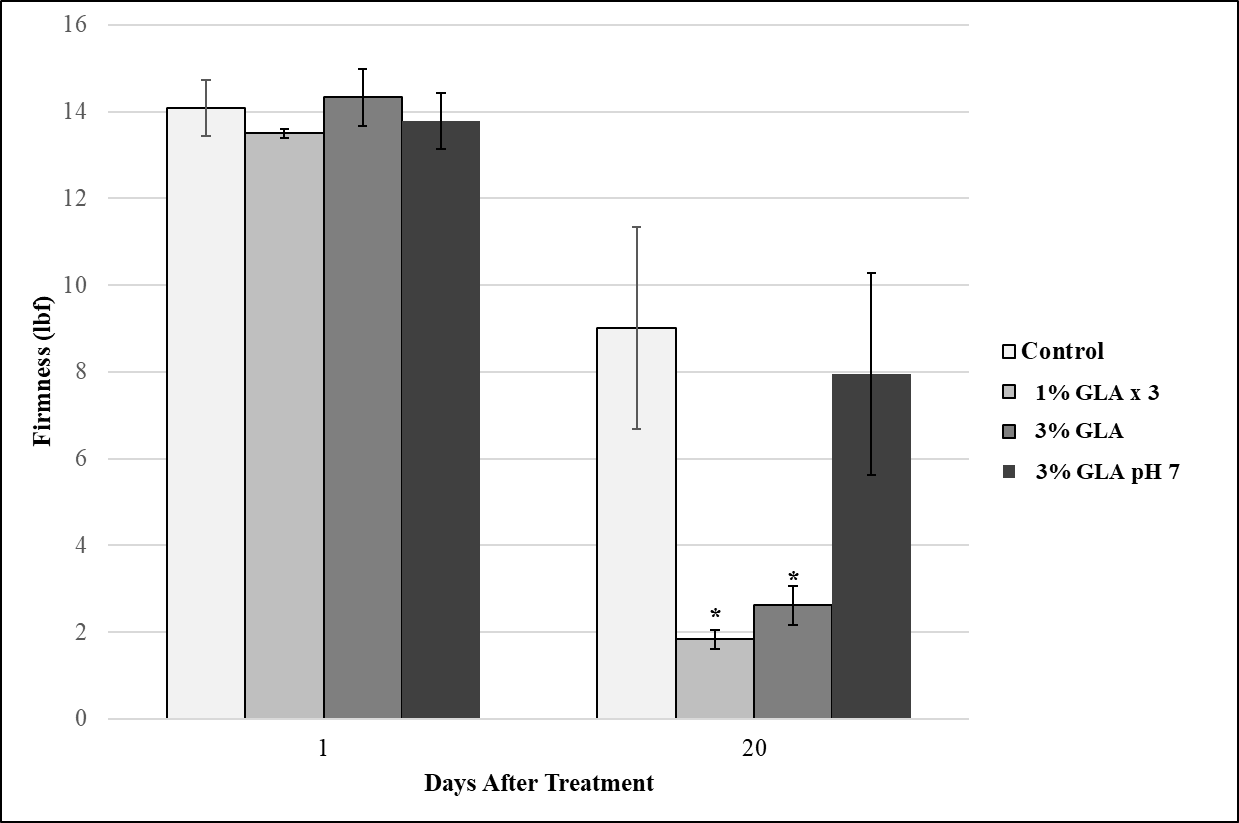


**Supplementary Figure 3.2a.** Starting and ending firmness of 1-MCP treated ‘D’Anjou’ pear fruit subjected to GLA treatment in May 2017. Asterisks indicate significant difference from the control (p<0.05).


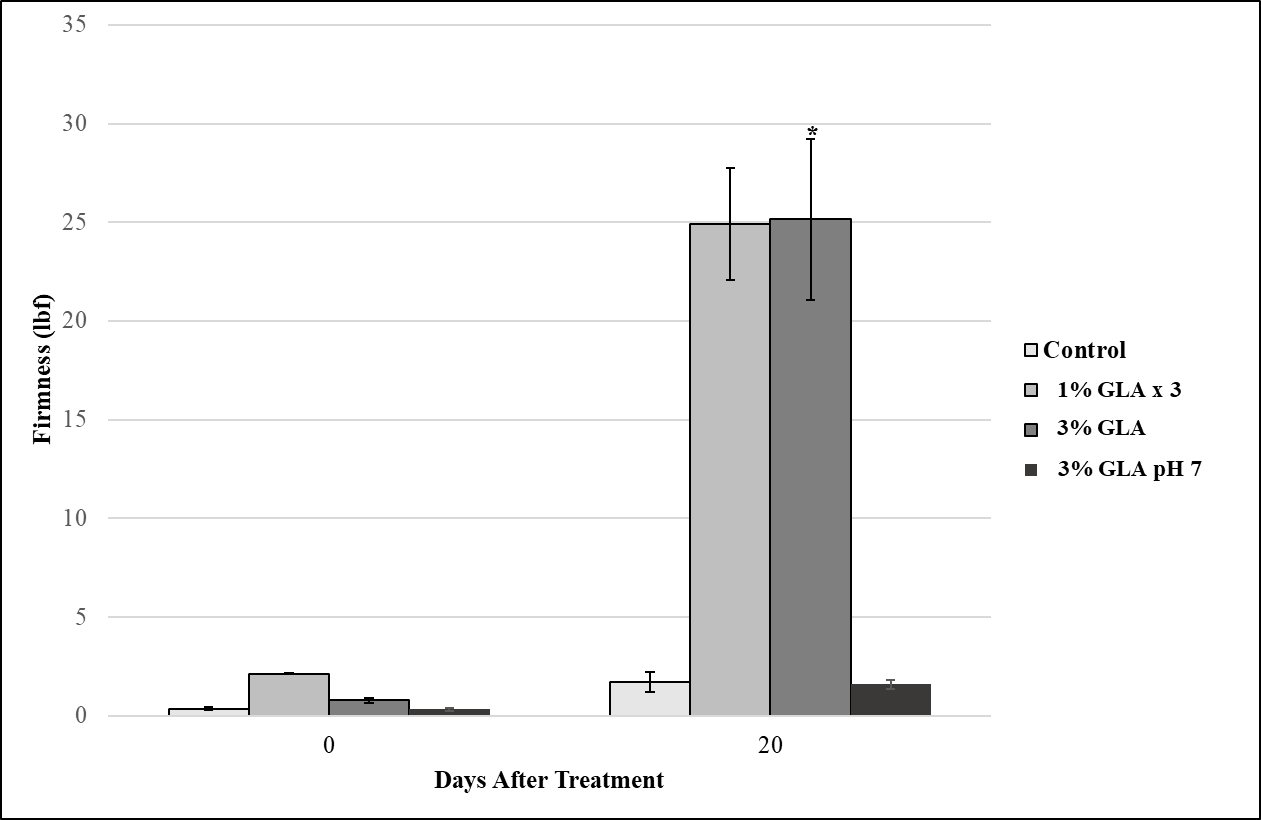


*****

*****

**Supplementary Figure 3.2b.** Internal ethylene concentrations of 1-MCP treated ‘D’Anjou pear fruit subjected to GLA treatments in May 2017. Asterisks indicate significant difference from the control (p<0.05).


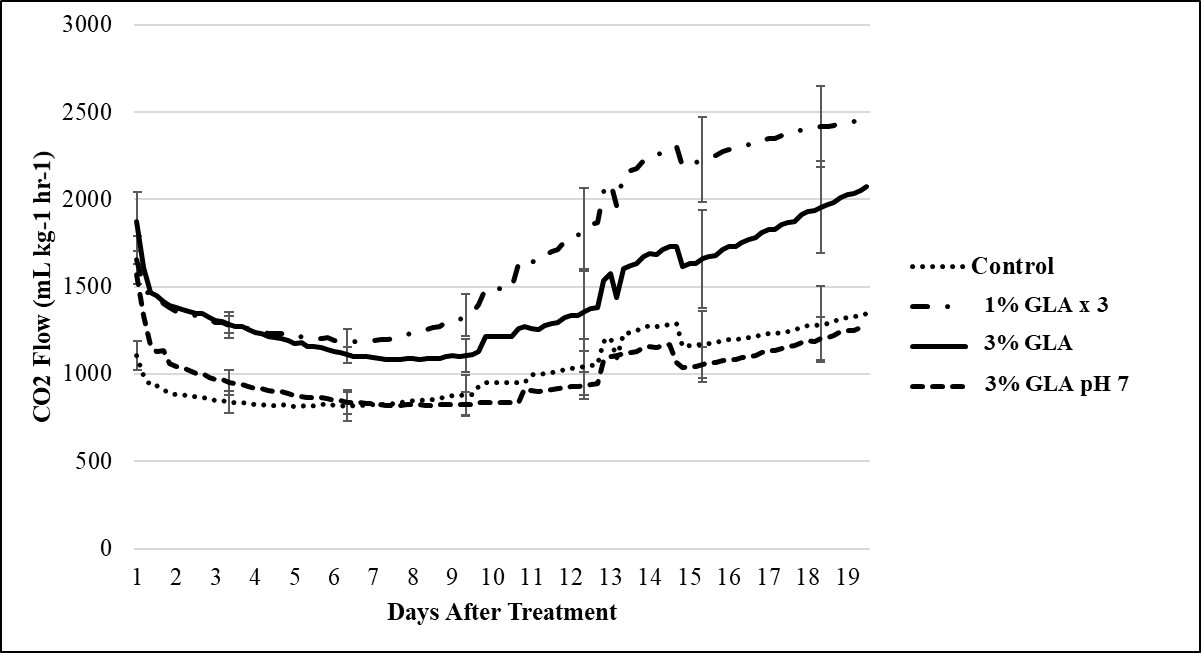


**Supplementary Figure 3.2c** Carbon dioxide evolution of 1-MCP treated ‘D’Anjou’ pears over the course of 19 days in May 2017. (Day 20 is not shown, because not all replicates of each treatment were measured before the experiment was terminated). Error bars represent standard error measurements calculated for days 0, 4, 7, 10, 13, and 16 following treatment with GLA ripening compound or control solutions. p<0.05 when comparing the 3% GLA and 1% GLA x3 treatments to the Control and 3% GLA titrated to neutral pH.

**Supplementary Figure 3.3a.** Starting and ending firmness of 1-MCP treated ‘D’Anjou’ pear fruit subjected to GLA treatments in June 2017.

**Supplementary Figure 3.3b.**  Internal ethylene evolution of 1-MCP treated ‘D’Anjou’ pear fruit subjected to GLA treatment in June 2017.

**Supplementary Figure 3.3c.** CO_2_ evolution (respiration) of 1-MCP treated ‘D’Anjou’ pear fruit subjected to GLA treatments in June 2017.

**Supplementary Figure 3.4a.** Firmness of 1-MCP treated ‘D’Anjou’ pear fruit subjected to GLA treatments in July 2017. Asterisks indicate significant difference from the control (p<0.05).

**Supplementary Figure 3.4b** Internal ethylene concentration of 1-MCP treated ‘D’Anjou’ pears following treatment with GLA in July 2017.

**Supplementary Figure 3.5a.** Firmness of 1-MCP treated ‘D’Anjou pears following treatment with 3% GLA at various pHs in January-February 2018.

**Supplementary Figure 3.5b.** Firmness of 1-MCP treated ‘D’Anjou’ pears following treatment with 3% GLA at various pHs in February 2018.

**Supplementary Figure 3.5c.** Firmness of 1-MCP treated ‘D’Anjou’ pears following treatment with 3% GLA at various pHs in June 2018.

**Supplementary Figure 3.6a.** Internal ethylene of 1-MCP treated ‘D’Anjou’ pears following treatment with 3% GLA at various pHs in January-February 2018.

**Supplementary Figure 3.6b.** Internal ethylene evolution of 1-MCP treated ‘D’Anjou’ pears following treatment with 3% GLA at various pHs in February 2018.

**Supplementary Figure 3.6c** Internal ethylene evolution of 1-MCP treated ‘D’Anjou’ pears following treatment with 3% GLA at various pHs in June 2018.

**Supplementary Figure 3.7a.** Soluble solid content of 1-MCP treated ‘D’Anjou’ pears following treatment with 3% GLA at various pHs in February 2018.

**Supplementary Figure 3.7b.** Soluble solid content of 1-MCP treated ‘D’Anjou’ pears following treatment with 3% GLA at various pHs in June 2018.

**References**

1. Kka, N., Rookes, J. & Cahill, D. Quantitation of ascorbic acid in Arabidopsis thaliana reveals distinct differences between organs and growth phases. *Plant growth regulation* **81**, 283-292 (2017).

2. Zommick, D.H., Knowles, L. & Knowles, N. Tuber respiratory profiles during low temperature sweetening (LTS) and reconditioning of LTS-resistant and susceptible potato (Solanum tuberosum L.) cultivars. *Postharvest biology and technology* **92**, 128-138 (2014).
