# Supplementary material for: Glyoxylic acid overcomes 1-MCP-induced blockage of fruit ripening in Pyrus communis L. var. ‘D’Anjou’
Source: Sci Rep. 2020 Apr 27;10:7084. doi: 10.1038/s41598-020-63642-z (PMC7184741; doi:10.1038/s41598-020-63642-z)
Supplement: Supplementary file 10 — Supplementary Information10. [file 41598_2020_63642_MOESM10_ESM.pptx]

## Slide 1
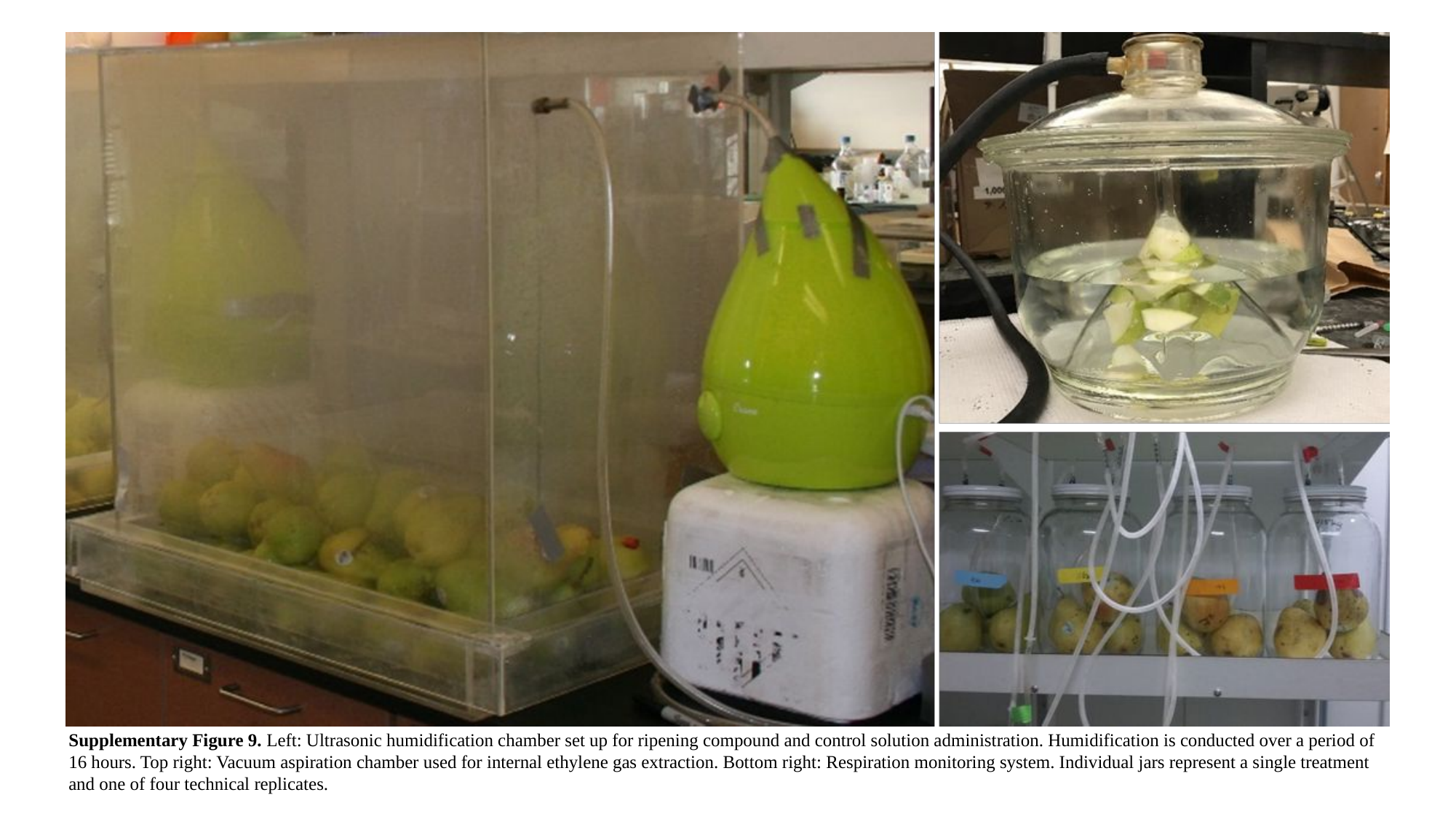

Supplementary Figure 9. Left: Ultrasonic humidification chamber set up for ripening compound and control solution administration. Humidification is conducted over a period of 16 hours. Top right: Vacuum aspiration chamber used for internal ethylene gas extraction. Bottom right: Respiration monitoring system. Individual jars represent a single treatment and one of four technical replicates.
